# Electroacupuncture to point Baihui confers anxiolytic effects by promoting oxytocin release from PVN in Mice

**DOI:** 10.1186/s13020-025-01307-7

**Published:** 2026-01-13

**Authors:** Hengxin Gong, Ban Feng, Ke Cheng, Runsong Cao, Ruifan Zhao, Zaihua Zhao, Ying-ying Zhang, Dake Song, Min Wang, Xu-bo Li, Yu-mei Wu, Shuibing Liu, Kun Zhang

**Affiliations:** 1 Department of Pharmacology, School of Pharmacy, Fourth Military Medical University, Xi’an, 710032 China; 2 State Key Laboratory of Oral & Maxillofacial Reconstruction and Regeneration, National Clinical Research Center for Oral Diseases, Shaanxi Engineering Research Center for Dental Materials and Advanced Manufacture, Department of Pharmacy, The Third Affiliated Hospital of the Fourth Military Medical University, Xi’an, 710032 China; 3 Shanghai Key Laboratory of Anaesthesiology and Brain Functional Modulation, Translational Research Institute of Brain and Brain-Like Intelligence, Clinical Research Center for Anaesthesiology and Perioperative Medicine, Department of Anaesthesiology and Perioperative Medicine, Shanghai Fourth People’s Hospital, School of Medicine, Tongji University, Shanghai, 200434 China; 4 Department of Pediatrics, Tangdu Hospital, Fourth Military Medical University, Xi’an, 710032 Shaanxi China; 5 Department of Occupational and Environmental Health and the Ministry of Education Key Lab of Hazard Assessment and Control in Special Operational Environment, School of Public Health, Fourth Military Medical University, Xi’an, 710032 Shaanxi China; 6 Precision Pharmacy & Drug Development Center, Department of Pharmacy, Tangdu Hospital, Fourth Military Medical University, Xi’an, 710038 China

**Keywords:** Anxiety-like behaviors, Paraventricular nucleus of the hypothalamus, Electroacupuncture, Oxytocin, Baihui (GV20)

## Abstract

**Background:**

Anxiety disorders—including generalized anxiety disorder, posttraumatic stress disorder, and social anxiety disorder—are highly prevalent psychiatric conditions that impose substantial clinical and social burdens. Preclinical and clinical studies have shown that electroacupuncture (EA) can effectively alleviate anxiety-like behaviors; however, the specific neural circuits and molecular mechanisms underlying EA’s therapeutic effects remain incompletely elucidated.

**Methods:**

We first assessed the impacts of EA at four classical acupoints—Zusanli (ST36), Neiguan (PC6), Tianshu (ST25), and Baihui (GV20)—delivered with distinct stimulation waveforms on anxiety-like behaviors in conventionally housed mice, using the elevated plus maze and open field test paradigms. To identify the neural circuit underlying the behavioral effects of Baihui (GV20) EA, we employed pseudorabies virus expressing enhanced green fluorescent protein (PRV-EGFP) for retrograde tracing from Baihui (GV20) and quantified c-Fos expression across the whole brain as a marker of neuronal activation. ELISA was utilized to measure plasma oxytocin (OXT) levels following EA at Baihui (GV20). Furthermore, a selective pharmacological antagonist of the oxytocin receptor (OXT-R) was administered to verify the critical role of OXT signaling in mediating the anxiolytic benefits of Baihui (GV20) EA.

**Results:**

EA at GV20 using intermittent electrical wave stimulation exhibited the most robust anxiolytic effects compared to EA at other acupoints or alternative stimulation parameters. Retrograde virus tracing from GV20 revealed a direct neuronal connection between the PVN and the GV20 acupoint region. Further experiments showed that GV20 EA significantly increased the activation of OXT-synthesizing neurons in the PVN and elevated peripheral OXT concentrations in mouse plasma. Critically, intraperitoneal injection of an OXTR antagonist completely abrogated the anxiolytic effects of GV20 EA, confirming that OXT signaling is indispensable for this therapeutic action.

**Conclusions:**

Intermittent 1.5 mA EA at Baihui (GV20) mitigates anxiety-like behavior in mice via a PVN-derived, OXT-dependent pathway. This work clarifies the anatomical and molecular mechanisms underlying EA-mediated anxiety relief and provides a basis for further exploring functional connections between specific acupoints and brain regions.

## Introduction

Anxiety disorders, such as generalized anxiety disorder, posttraumatic stress disorder, and social anxiety disorder, are among the most common psychiatric illnesses with a lifetime prevalence of approximately 30% [1]. These disorders frequently co-occur with other mental health issues, such as depression and insomnia, complicating clinical management and treatment [2]. Despite their substantial individual and societal burden, anxiety disorders remain widely underdiagnosed and inadequately treated across populations worldwide [3]. Although selective serotonin reuptake inhibitors and serotonin-norepinephrine reuptake inhibitors are recommended as first-line treatment drugs for anxiety [4], their clinical application is limited due to associated risks, including the potential to trigger epilepsy, increase anxiety and tension, and induce other adverse effects [5, 6]. However, treatment of anxiety disorders generally achieves only partial remission of symptoms or shows a high rate of relapse [7], highlighting the need for safer therapeutic options [8]. In particular, certain non-pharmacological physical interventions—such as exercise and music therapy—have garnered considerable attention [9, 10].

Acupuncture has been used in China for over 2,500 years. Electroacupuncture (EA) refers to the application of a pulsating electrical current to acupuncture needles for acupoints, and is a form of acupuncture therapy that has been widely used in clinical and scientific research [11, 12]. Clinically, acupuncture and EA are effective in treating anxiety on their own or as adjuncts to pharmacological therapy [13]. Furthermore, in a randomized clinical trial of EA treatment for insomnia in patients with depression, quality of sleep improved significantly in the EA group [14]. In mouse models of spared nerve injury or chronic inflammatory pain, EA also showed significant anxiolytic effect [15, 16]. Although several brain regions—including the rostral anterior cingulate cortex (rACC) [17], ventral hippocampus (vHPC) [18], and basolateral amygdala (BLA) [19]—have been implicated in the neural circuits mediating the beneficial effects of EA, the specific neural circuits and underlying molecular mechanisms remain to be fully elucidated.

Recently, neuropeptides have emerged as viable candidates, such as oxytocin (OXT) [20], arginine vasopressin [21], neuropeptide Y [22], and neuropeptide S [23]. As a neuropeptide synthesized by the hypothalamus, OXT is a neurohypophyseal nonapeptide primarily synthesized in the hypothalamus and is highly evolutionarily conserved [24]. OXT exerts functions by binding to the oxytocin receptor (OXTR), a seven-transmembrane G-protein-coupled receptor. OXT neurons restrictively located in the paraventricular nucleus of the hypothalamus (PVN), supraoptic and accessory nuclei of the hypothalamus in the brain, while widely spread projections in the brain, and the distribution of OXTR within the brain is also widespread [25]. Accumulating evidence suggests that the OXT/OXTR system plays important roles in anxiety and depression [26]. It has been shown that the hypothalamic OXT/OXTR system contributes to the regulation of the stress response [27]. A study found that the mRNA level of OXTR in the blood positively correlated with the hyporesponsive hypothalamic–pituitary–adrenal (HPA) axis subtype of posttraumatic stress disorder patients [28]. Intracerebroventricular OXT administration attenuated both the neuroendocrine and molecular responses of the HPA axis in restraint-stressed rats [29]. Thus, these previous studies suggested that the hypothalamic OXT/OXTR system plays an important role in anxiolytic effects.

In this study, we investigated the effects of EA at different acupoints, namely Zusanli (ST36), Neiguan (PC6), Tianshu (ST25), and Baihui (GV20), on anxiety-like behaviors in normally housed mice. Our findings revealed that EA applied at Baihui (GV20) with interrupted electric wave stimulation exhibited the most pronounced anxiolytic effects. Anatomical tracing using retrograde pseudorabies virus expressing enhanced green fluorescent protein (PRV-EGFP), injected at Baihui (GV20), demonstrated a neuronal connection between the PVN and Baihui (GV20). Subsequent experiments showed that Baihui (GV20) EA stimulation increased the activation of OXT-producing neurons in the PVN and elevated peripheral OXT levels in the plasma. Intraperitoneal injection of an OXTR antagonist completely abolished the anxiolytic effects of EA, indicating a critical role for OXT signaling. Collectively, our findings identify a novel PVN OXT neuron-dependent pathway that mediates the anxiolytic effects of EA at Baihui (GV20), thereby providing critical mechanistic insights into the therapeutic potential of acupuncture for anxiety-related disorders.

## Materials and methods

### Animals

Adult C57BL/6 J mice (8 weeks old) were used in the experiments, with an equal number of females and males in each group to reduce potential gender-related differences. Mice were housed in groups of up to 5 per cage under a 12-h light/dark cycle, with free access to food and water. All animal procedures were reviewed and approved by the Institutional Animal Care and Use Committee of the Fourth Military Medical University (FMMU, approval No. IACUC-20230115). All tests were performed in a double-blinded manner, and animals were randomly assigned to experimental groups using a random number generator. For mice undergoing surgery, suturing was followed by administration of penicillin sodium (approximately 200 U/g). Postoperatively, the animals were placed on a heating pad to maintain body temperature until they regained consciousness. Operated mice were then housed individually in plastic cages, with ad libitum access to food and water in the colony room. Every effort was made to minimize the number of animals sacrificed in the experiments.

### EA Intervention

The mice were anesthetized with 1–2% isoflurane mixed in oxygen. Acupuncture needles (0.16 × 7 mm in diameter) were inserted to a depth of 3–4 mm at the following bilateral acupoints: Zusanli (ST36): 5 mm lateral to the anterior tubercle of the tibia; Neiguan (PC6): 2 mm proximal to the wrist crease, between the tendons of the palmaris longus and flexor carpi radialis; Tianshu (ST25): 5 mm lateral to the anterior midline, at the junction of the upper two-thirds and lower one-third of the line connecting the xiphoid process and the superior border of the pubic symphysis. For Baihui (GV20) stimulation—located at the intersection of the skull’s midline and the line connecting the tips of both ears—one needle was inserted obliquely posteriorly at a 15° angle to a depth of 3–4 mm, and an additional needle was placed 3 mm adjacent to GV20 using the same insertion parameters.

In the sham EA group, needles were inserted subcutaneously at GV20 without electrical stimulation. All other groups received isoflurane anesthesia following the same protocol as the EA and sham EA groups, with the total observation period matching that of the EA group. The needles were connected to an acupoint nerve stimulator (CMNS6-2, Wuxi Jiajian Medical Instrument Co., Ltd., Wuxi, China) for a 15-min stimulation session. The intervention parameters were as follows: Continuous Wave: 10 Hz frequency, 1.5 mA stimulating intensity; Interrupted Wave: 10 Hz frequency, 1.5 mA stimulating intensity with a 5-s on-time followed by a 3-s off-time; Dense-Sparse Wave: Alternating between a sparse wave (10 Hz) and a dense wave (20 Hz), 1.5 mA stimulating intensity, with each phase lasting 1.5 s. To minimise observer bias, a strict double-blinding strategy was implemented. Two independent researchers were involved: (1) A “treatment allocator” (unblinded to group assignments) prepared EA devices with pre-set parameters (intensity; waveform) or sham EA (identical device without electrical output) and labeled cages with cryptic codes. (2) A “treatment administrator” (blinded to EA/sham status and experimental hypotheses) performed the stimulation according to the coded labels.

### Drug administration

L-368,899 (catlog. HY-10867, MedChemExpress, China), a selective OXTR antagonist, was dissolved in saline and administered intraperitoneally (1 mg/kg) 30 min before EA [30, 31]. Same volume of saline was injected in the control mice. The researchers responsible for animal injection, behavioral testing, and tissue collection were blinded to reagent identities and group allocations.

### Elevated plus maze (EPM) test

The EPM apparatus consisted of a central platform (6 × 6 cm) with two opposing closed arms (30 × 6 × 15 cm) and two opposing open arms (30 × 6 cm), forming a plus-shaped structure. The entire apparatus was positioned 40 cm above the floor in a quiet, dimly lit room. Each mouse was individually placed on the central platform, facing an open arm, and allowed to explore the maze freely for 5 min. Mouse behavior was recorded via a overhead camera, and the duration spent in the open arms—an indicator of anxiety levels—was analyzed using JLBehv software (Shanghai Jiliang Software Technology Co., Ltd.). Between trials, the maze was thoroughly cleaned with 75% ethanol to eliminate residual olfactory cues.

### Open-field test (OFT)

Mice were gently placed at the center of a cubic chamber (40 × 40 × 40 cm) at the start of the test and permitted to explore freely for 5 min. Movements were recorded and analyzed using JLBehv software (Shanghai Jiliang Software Technology Co., Ltd.). Anxiety-like behavior was evaluated by measuring the time spent in the central zone (20 × 20 cm) of the chamber, while total distance traveled was used to assess locomotor activity. All mice were habituated to the testing room for 12 h prior to the OFT. The testing room was maintained with dim, indirect white lighting. After each trial, the open-field chamber was cleaned with 75% ethanol to remove residual olfactory cues.

### Viral injection

For retrograde tracing of the Baihui (GV20) circuit, naive mice received unilateral injections of PRV-EGFP (3.2 × 10^12^ vg/mL, 1 μL, cat. no. BC-PRV-531, Braincase (Shenzhen) Biotechnology Co., Ltd., China) into the Baihui (GV20) acupoint. Injections were administered using a Hamilton syringe at a 15° angle to a depth of 3–4 mm, with an infusion rate of 100 nL/min. After each injection, the needle was left in place for an additional 8–10 min to promote viral diffusion before being slowly withdrawn. Mice were returned to their home cages following full recovery from anesthesia in a 37 °C incubator. Three days after injection, the mice were anesthetized and euthanized for brain sample collection.

### Enzyme‐linked immunosorbent assay (ELISA)

ELISA was used to quantify OXT levels in plasma and the PVN using an ELISA kit (Catalog No: E-EL-0029, Elabscience) following the manufacturer’s instructions. Briefly, PVN tissues were dissected and homogenized on ice using a homogenizer in lysis buffer containing 25 mM HEPES (pH 7.4), 0.1% 3-[(3-cholamidopropyl) dimethylammonio]-1-propanesulfonate, 5 mM MgCl₂, 1.3 mM EDTA, 1 mM EGTA, 10 μg/ml pepstatin, aprotinin, leupeptin, and 1 mM phenylmethylsulfonyl fluoride (PMSF). The homogenates were centrifuged at 10,000 × *g* for 15 min, and the supernatants were collected for ELISA analysis.

### Immunofluorescence staining

Brain slices with PVN were prepared as previously described [32]. Briefly, brain slices were permeabilized with 0.3% Triton X-100 for 15 min, then blocked with QuickBlock™ (Beyotime, China) for 1 h. Subsequently, the 20-μm thick slices were incubated with primary antibodies overnight at 4 °C in a refrigerator. For single immunofluorescence staining, rabbit anti-c-FOS (1:1000, Cell Signaling Technology, USA, catlog. #2250) was used. For double immunofluorescence staining, the following primary antibody combinations were employed: mouse anti-Neurophysin I (1:1000, Santa Cruz Biotechnology, USA, catlog. sc-393907) with rabbit anti-c-FOS (1:1000, Cell Signaling Technology, USA). After washing the slices with PBS containing 0.3% Triton X-100, the following secondary antibodies were added and incubated at room temperature for 2 h: Alexa 594-conjugated goat anti-rabbit antibodies (1:1000, Life Technologies Corporation, USA) and Alexa 488-conjugated goat anti-rabbit antibodies (1:1000, Life Technologies Corporation, USA). Nuclei were stained with DAPI. Images were captured using a fluorescence microscope (BX43, Olympus, Japan) and analyzed with ImageJ software.

### Statistical analysis

All the data are shown as the mean ± standard deviation (SD). Statistical analysis was carried out using SPSS 21.0 software. The significance between two groups was analyzed using one‐way ANOVA followed by Tukey‐Kramer multiple comparisons test or Student’s unpaired *t* test. Before statistical analysis, the data were tested for Gaussian distribution assessed using Shapiro–Wilk normality test after transform to logarithms. *p* < 0.05 was considered to indicate a statistically significant difference.

## Results

### EA at Baihui (GV20) significantly alleviated anxiety‐like behaviors in normally housed mice

**Fig. 1:**
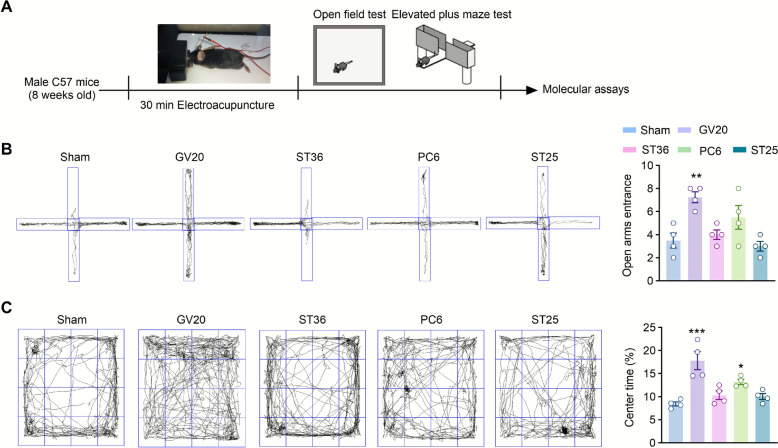
EA treatment at Baihui (GV20) decreased anxiety-like behaviors. **A** Experimental design and timeline for EA treatment. **B** Representative movement trajectories and open arms entrance of different groups mice in the elevated plus maze test. **C** Representative movement trajectories and center time ratio of different groups mice in the open field test. n = 4 mice. Data are presented as the Mean ± SEM. *p < 0.05, **p < 0.01, ***p < 0.001 vs. Sham

Based on previous research findings, our study selected several commonly used acupuncture points, including Baihui (GV20), Neiguan (PC6) [14], Zusanli (ST36) [15], and Tianshu (ST25) [33]. The experimental process was carried out as follows: First, the mice were anesthetized with isoflurane. After ensuring the anesthesia took effect, electroacupuncture (EA) was administered to the mice for 30 min. Immediately after the EA intervention, the elevated plus maze test (EPM) and open field test (OFT) were conducted to evaluate the mice's anxiety-like behaviors (Fig. 1A). The test results showed that after EA stimulation at different acupuncture points, the mice that received EA at Baihui (GV20) exhibited the least anxiety-like behaviors. Specifically, in the EPM, these mice spent significantly more time in the open arms (Fig. 1B); in the OFT, they also spent significantly more time in the center zone (Fig. 1C). These consistent results from both behavioral tests strongly indicated that Baihui (GV20) is the most effective acupuncture point for EA stimulation to produce anxiolytic effects.

### EA at Baihui (GV20) with appropriate intensity and a dense-sparse wave demonstrated the most pronounced anxiolytic efficacy

**Fig. 2:**
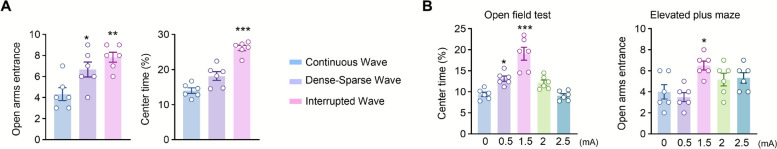
EA treatment with dense-sparse wave, 1.5 mA showed the most pronounced anxiolytic effects. **A** Performance of mice receiving electroacupuncture (EA) with different waveforms (i.e., continuous wave, dense-sparse wave, and interrupted wave) in the elevated plus maze test and open field test. **B** Performance of mice receiving electroacupuncture (EA) with different intensities (i.e., 0, 0.5, 1.5, 2, and 2.5 mA) in the elevated plus maze test and open field test. n = 4 mice. Data are presented as the Mean ± SEM. *p < 0.05, **p < 0.01, ***p < 0.001 vs. continuous wave or 0 mA

The therapeutic performance of EA is intricately regulated by several key parameters, including waveform, frequency, and stimulation intensity, among which waveform characteristics have been identified as a primary determinant of treatment efficacy. In contemporary EA research and clinical practice, three waveforms are most commonly employed: the continuous wave, the dense-sparse wave and interrupted wave. To systematically optimize EA parameters for anxiety management, our study implemented a two-phase investigation focusing on waveform and intensity at the Baihui (GV20) acupoint. In the initial phase, we compared the anxiolytic effects of different waveforms. As illustrated in Fig. 2B, the dense-sparse wave consistently outperformed other tested waveforms, demonstrating superior efficacy in reducing anxiety-like behavioral indicators. Recognizing that stimulation intensity is another critical variable influencing EA outcomes, the second phase of our research evaluated four distinct intensity levels: 0.5 mA, 1.5 mA, 2 mA, and 2.5 mA. Through rigorous behavioral assessments, we observed that the 1.5 mA intensity elicited the most favorable responses, as evidenced by significant reductions in anxiety-related behaviors compared to the other tested intensities. Notably, lower intensities (0.5 mA) failed to produce sufficient physiological stimulation, while higher intensities (2 mA and 2.5 mA) showed diminished efficacy, potentially due to excessive neural excitation or discomfort-induced stress responses. Taken together, these findings conclusively demonstrate that the combination of a dense-sparse wave and a stimulation intensity of 1.5 mA constitutes the optimal EA parameter configuration at Baihui (GV20) for alleviating anxiety-like behaviors.

### Baihui (GV20) is innervated by the PVN, and EA stimulation at Baihui (GV20) enhances PVN activation

**Fig. 3:**
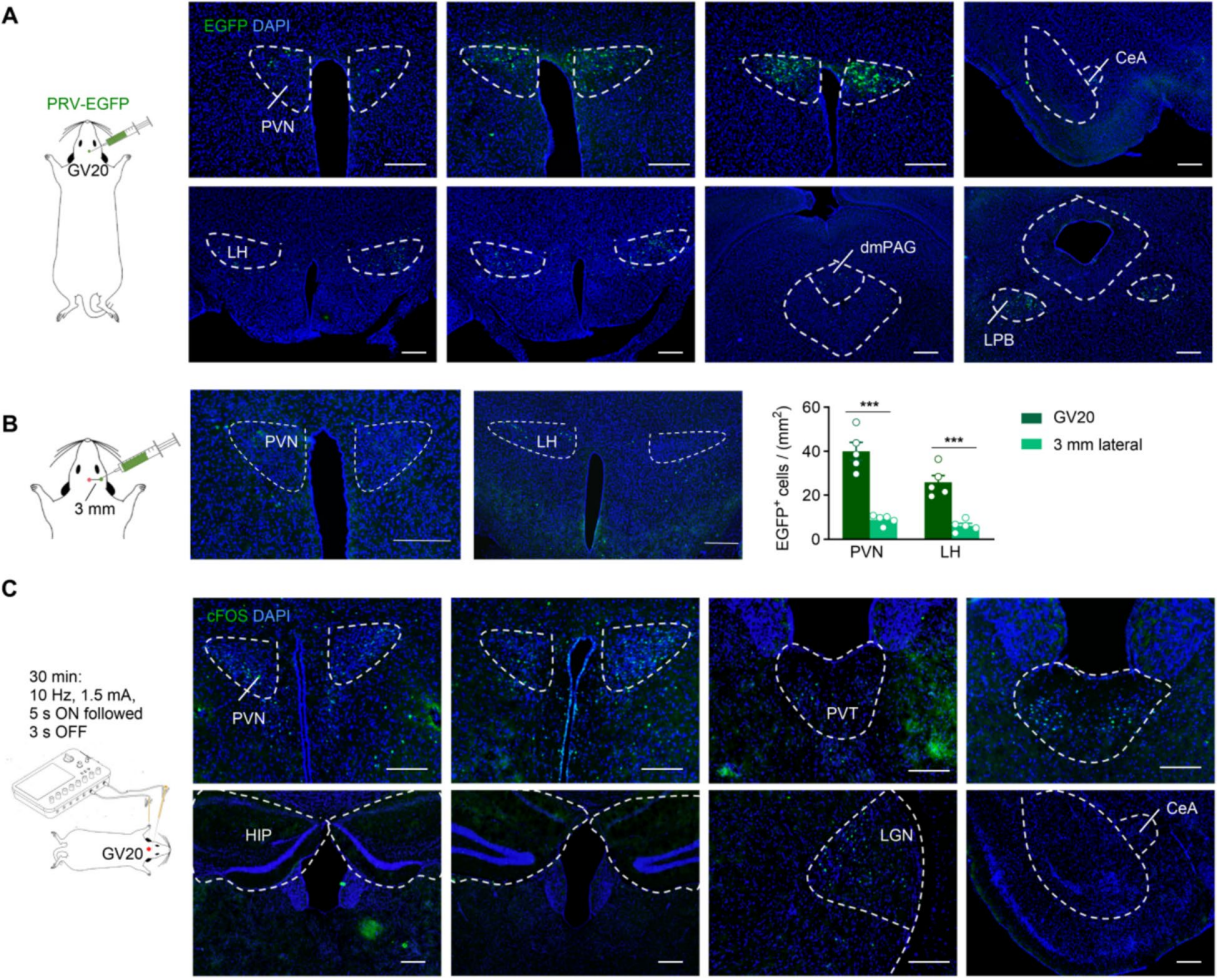
PVN was anatomically connected with Baihui (GV20) and was activated by EA at this acupoint. **A** Schematic with pseudorabies virus expressing enhanced green fluorescent protein (PRV-EGFP) injection in Baihui (GV20) and representative images of showing viral EGFP expression in some brain areas. **B** Schematic diagram illustrating PRV-EGFP injection at the site 3 mm lateral to Baihui (GV20) acupoint, representative micrographs showing viral EGFP expression in selected brain regions, and statistical analysis of EGFP-positive (EGFP^+^) cell counts in the PVN and LH. n = 5 mice. Data are presented as the Mean ± SEM. ***p < 0.001. **C** Representative images showing the c-Fos signals in the different brain areas after EA at Baihui (GV20) for 30 min. PVN, paraventricular nucleus of the hypothalamus; CeA, central amygdala; LH, lateral hypothalamus; dmPAG, dorsomedial periaqueductal gray; LPB, lateral parabrachial nucleus; PVT, paraventricular thalamus; LGN, lateral geniculate nucleus; HIP, hippocampus. Scale bar = 200 μm

Numerous studies have demonstrated that the beneficial effects of electroacupuncture (EA) at specific acupoints are mediated by the nervous system [34, 35]. To further delineate the neural circuits underlying the therapeutic actions of EA at Baihui (GV20), we employed a retrograde tracing approach. Pseudorabies virus (PRV) is extensively employed in neuroanatomical investigations owing to its distinctive retrograde transneuronal tracing properties, which enable the precise cross-synaptic mapping of neural circuitry [36, 37]. In our experiments, a recombinant pseudorabies virus expressing enhanced green fluorescent protein (PRV-EGFP) was stereotactically microinjected into the Baihui acupoint (GV20). By leveraging the virus’s retrograde labeling capacity, this strategy allowed us to identify neurons that are putatively responsible for the primary innervation of the Baihui acupoint (GV20). We observed EGFP-labeled cells associated with Baihui (GV20) in several key brain regions, including the paraventricular nucleus of the hypothalamus (PVN), central amygdala (CeA), lateral hypothalamus (LH), dorsomedial periaqueductal gray (dmPAG), and lateral parabrachial nucleus (LPB) (Fig. 3A). In contrast, when PRV-EGFP was microinjected into the site 3 mm lateral to the Baihui (GV20) acupoint, the number of EGFP-labeled cells was significantly lower than that following microinjection into the Baihui (GV20) acupoint itself (Fig. 3B). These findings clearly delineate the central neural connections of the Baihui (GV20) acupoint, thereby providing an anatomical foundation for understanding its neural regulatory network.

Subsequently, we aimed to investigate whether EA at Baihui (GV20) could modulate the activation of these brain regions. To assess neuronal activation, we utilized c-Fos immunostaining, a well-established marker for immediate early gene expression that reflects recent neuronal activity. Following EA intervention, our results revealed significant activation in the PVN, paraventricular thalamus (PVT), and lateral geniculate nucleus (LGN). In contrast, no notable increases in c-Fos expression were observed in the hippocampus (HIP) or central amygdala (CeA) (Fig. 3C). This indicates that the signal conduction pathway of acupoint acupuncture is closely related to the neural pathway. 

Taken together, these data indicate that among the brain regions connected to Baihui (GV20), the PVN is selectively activated by EA at Baihui (GV20), suggesting that it may serve as a critical target brain region mediating the beneficial effects of EA at this acupoint.

### EA at Baihui (GV20) exerting its anxiolytic effects by promoting oxytocin release from PVN

**Fig. 4:**
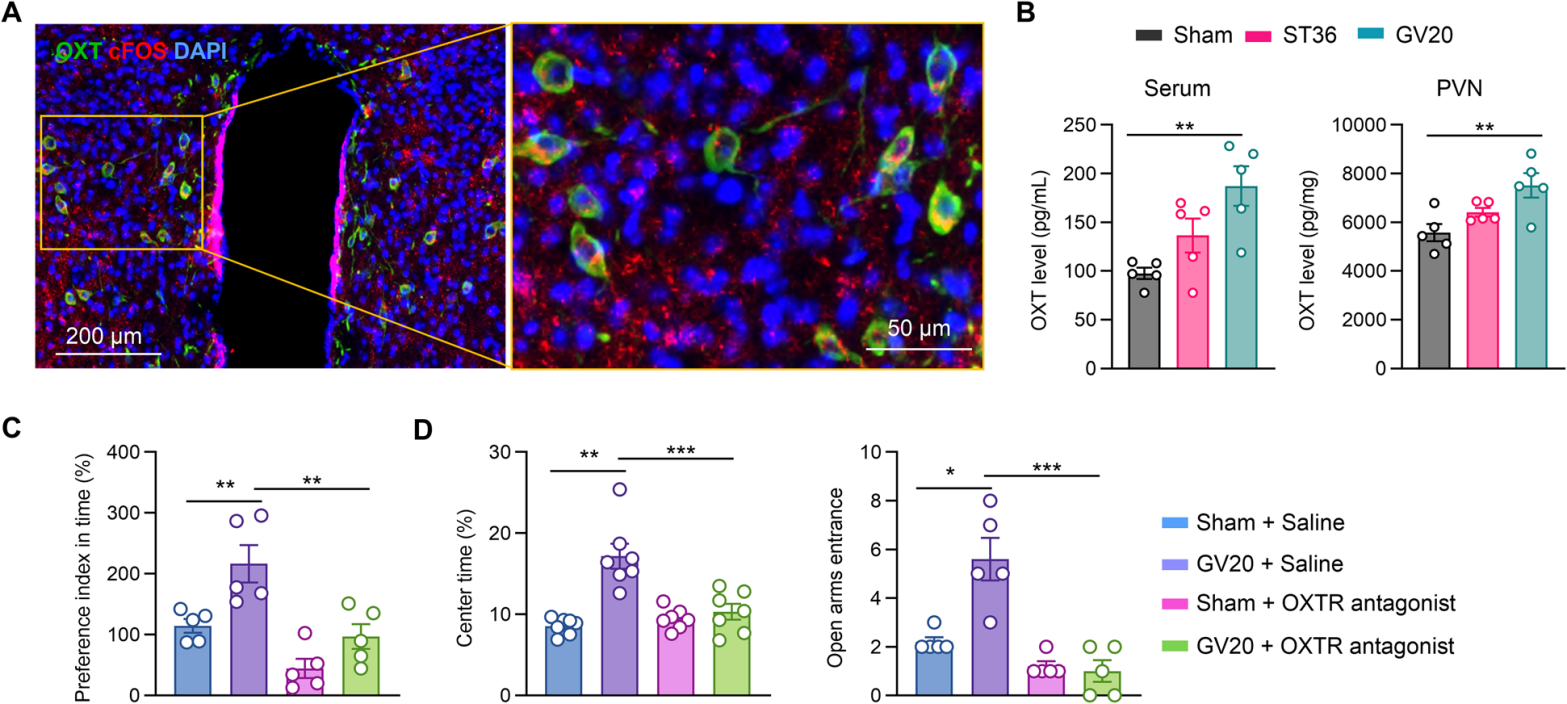
OXTR antagonist blocks the anxiolytic effect of EA at Baihui (GV20). **A** Representative images showing co-staining of c-FOS (red) and oxytocin (OXT, green) in the PVN of the hypothalamus following EA at Baihui (GV20). **B** Serum and PVN OXT levels after EA at Baihui (GV20). **C** Effects of OXTR antagonist pretreatment and EA at Baihui (GV20) on social behaviors as assessed by the social interaction test. **D** Effects of OXTR antagonist pretreatment on the anxiolytic effect of EA at Baihui (GV20), as assessed by the elevated plus maze test and open field test. n = 5 mice. Data are presented as the Mean ± SEM. *p < 0.05, **p < 0.01, ***p < 0.001

Given that the PVN acts as a pivotal integrative hub in orchestrating both the neuroendocrine arm and the autonomic nervous system component of the stress response—characterized by sympathetic nervous system activation and concurrent parasympathetic nervous system inhibition—our research focused on investigating the PVN as a key target. Moreover, OXT released from the PVN has been shown to exhibit significant anti-stress effects. Additionally, we observed that a substantial number of OXT-positive neurons were activated following EA stimulation at Baihui (GV20) (Fig. 4A). Building on these observations, we formulated the hypothesis that EA at Baihui (GV20) exerts its anti-stress effects by modulating OXT release from the PVN. To validate this hypothesis, we measured OXT levels in the serum and PVN following EA intervention. Our findings demonstrated that EA at Baihui (GV20)—but not at Zusanli (ST36)—significantly elevated OXT levels in both the PVN and serum (Fig. 4B), thereby providing initial evidence in support of our hypothesis. Concurrently, we conducted behavioral assessments using the social interaction test, as well as the OFT and EPM—two well-established paradigms for evaluating anxiety-like behaviors. Thirty minutes prior to EA treatment, administration of the OTR antagonist L368,899 hydrochloride (1 mg/kg, intraperitoneal [i.p.] injection) completely abrogated the EA-induced enhancement of social interaction duration in the social interaction test (Fig. 4C). Similarly, this antagonist reversed the EA-mediated elevation in center time in the OFT and the number of entries into the open arms in the EPM (Fig. 4D). Collectively, these results indicate that EA at Baihui (GV20) exerts its anxiolytic effects through an OXT-dependent pathway, underscoring the critical role of OXT in mediating the anti-stress actions of EA at this acupoint.

## Discussion

Acupuncture has been used in Traditional Chinese Medicine as a therapeutic intervention for the past 3000 to 4000 years, with the first codex formalizing acupuncture treatments—The Yellow Emperor’s Classic of Internal Medicine—published around 100 BC [38]. Currently, acupuncture is utilized worldwide for treating various medical conditions [35], particularly in the treatment of neuropsychiatric disorders including depression [39–41] and anxiety. Specifically, six weeks of acupuncture at acupoints Baihui (GV20) and Zusanli (ST36), or at Taichong (LR3), Sanyinjiao (SP6), Neiguan (PC6), and Shenmen (HT7), has been found to be as effective as oral fluoxetine in treating depression, while demonstrating superior response and improvement rates [42]. Similarly, acupuncture at ST36 and CV4 acupoints has shown efficacy in alleviating depressive-like behaviors in animal models [39, 42]. In the present study, we compared the effectiveness of EA at at different acupoints, including Baihui (GV20), Neiguan (PC6), Zusanli (ST36), and Tianshu (ST25), in ameliorating anxiety-like behaviors. We reported EA at Baihui (GV20) showed the best efficiency. Previous researches also indicated acupuncture at this acupoint improves cognitive function of APP/PS1 Alzheimer’s disease mice [43], modulates the brain-derived neurotrophic factor secretion in the hippocampal dentate gyrus [44].

As different stimulation patterns and intensities of sensory input may induce distinct effects [45], we tested various waveforms, including continuous, dense-sparse, and interrupted waves. Our findings indicate that the dense-sparse wave at 1.5 mA—characterized by alternating rapid (dense) and slower (sparse) electrical pulses—exerts the most pronounced beneficial effects. This rhythmic pulse alternation induces synchronized muscle contractions and relaxations, a mechanism demonstrated to enhance microcirculation, mitigate tissue edema, and elicit robust analgesic responses [46, 47]. These foundational effects underscore the potential of the dense-sparse wave in modulating neurophysiological pathways relevant to anxiety, though its underlying mechanism remains to be elucidated.

EA is thought to activate specific brain regions via sensory input. Baihui (GV20) may have anatomical connections with specific brain areas. In the present study, we employed PRV, a retrograde neural tracing tool, to anatomically validate a polysynaptic neuronal pathway connecting Baihui (GV20) and the PVN. While PRV tracing revealed that the Baihui (GV20) acupoint is innervated by the PVN, anterograde viral tracing more effectively delineates how somatosensory signals from Baihui (GV20) stimulation are transmitted to the brain via ascending pathways to regulate emotion. To map the nuclei involved in somatosensory neural networks, an efficient polysynaptic anterograde tracer with low toxicity is required. Currently, the most common neurotropic viral tool for anterograde polysynaptic tracing is herpes simplex virus (HSV) [48]. However, HSV rarely spreads from peripherally injected sites to the central nervous system [49]. Despite the fact that sensory and motor information can be processed by distinct neuronal populations, neuroanatomical evidence supports the existence of direct sensory-motor feedback in numerous brain regions [50, 51]. Thus, the utilization of PRV still holds significance to some extent.

Among the brain regions visualized via PRV injection, the PVN exhibited the highest density of EGFP-positive cells. The PVN contains two main types of neurons: magnocellular and parvocellular neurons. Magnocellular neurons in the PVN synthesize OXT and vasopressin, which are transported through the neurohypophysial tract to the median eminence. In contrast, parvocellular neurons project synapses to the junctional region of the median eminence, where they synthesize corticotropin-releasing hormone that is released into the hypophyseal portal vessels [52]. Additionally, parvocellular neurons send descending projections to the brainstem (particularly the nucleus of the solitary tract) and the intermediolateral cell column of the thoracolumbar spinal cord, thereby regulating sympathetic nervous system activation.

As a pivotal acupoint in traditional Chinese medicine (TCM), Baihui (GV20) is situated at the vertex of the cranium, precisely at the intersection of the sagittal midline of the skull and the line connecting the highest points of the two auricles. According to TCM theory, Baihui (GV20) belongs to the Governor Vessel (Du Meridian), which is designated as the “sea of Yang meridians.” In contrast, the PVN-OXT pathway—a well-established structural and functional substrate for the modulation of mental activities in modern neuroscience—can be interpreted through core TCM paradigms including Zangxiang (visceral manifestation), Jingluo (meridians and collaterals), and Hexie (harmony). This cross-disciplinary integration not only enriches the theoretical connotation of TCM but also endows it with a robust modern scientific foundation. OXT exerts numerous crucial psychological and physiological effects, including promoting social interaction, facilitating bonding, enhancing well-being, and reducing fear, stress, and pain [53]. However, whether stimulation of Baihui (GV20) preferentially activates OXT-positive parvocellular neurons remains to be further investigated. Additionally, emotional disorders can also be induced by brain-localized or systemic inflammatory and oxidative responses [54, 55]; thus, whether OXT exerts its anxiolytic effects by exerting anti-inflammatory and antioxidant activities across various organs requires further elucidation [56].

**Fig. 5:**
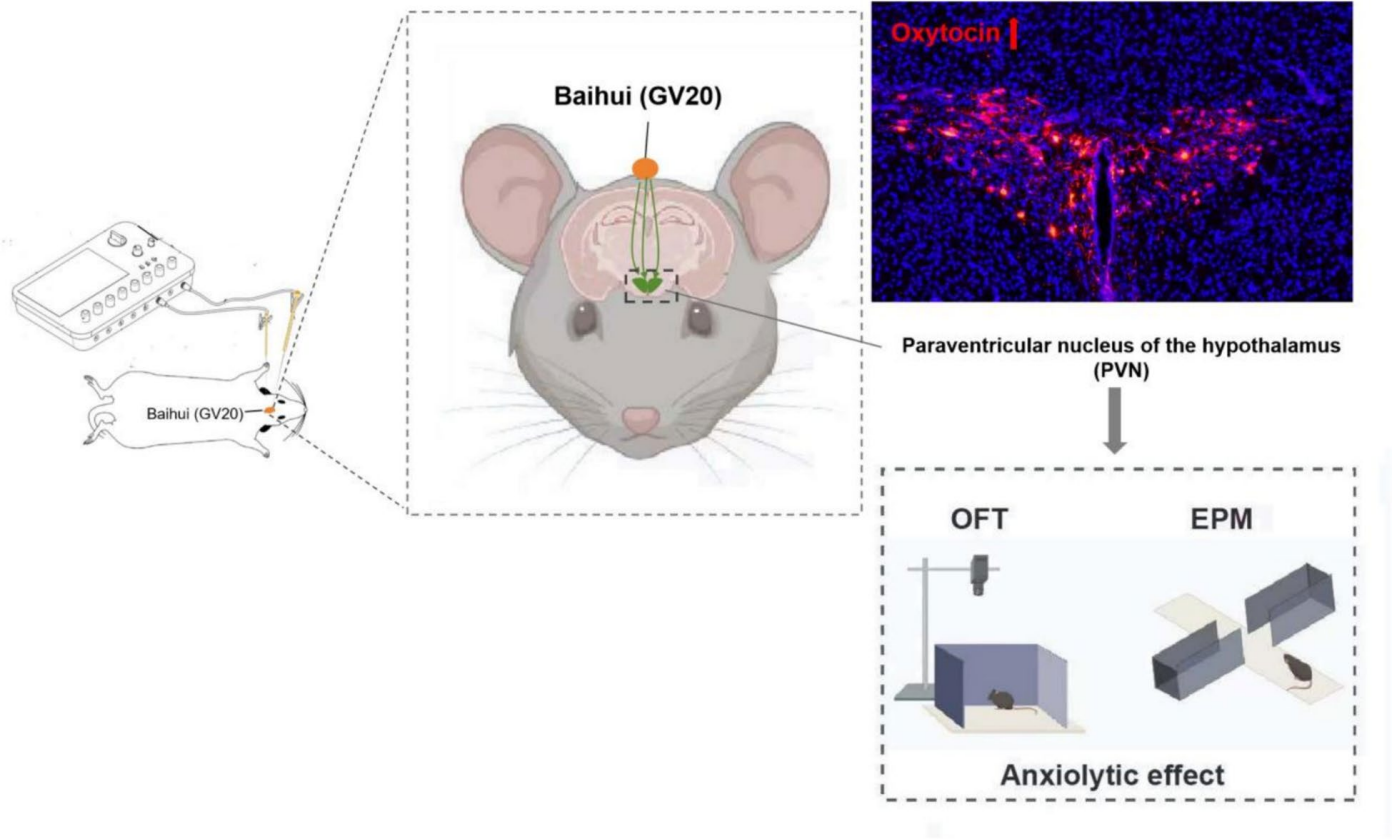
A direct neuronal connection between the hypothalamic paraventricular nucleus (PVN) and the Baihui (GV20) acupoint region was identified. Furthermore, electroacupuncture (EA) stimulation at Baihui (GV20) significantly enhanced the activation of oxytocin (OXT)-synthesizing neurons in the PVN and elevated circulating peripheral OXT concentrations in mouse plasma—effects that collectively contribute to its anxiolytic action

In conclusion, our study demonstrates that EA treatment at Baihui (GV20) with a sparse-dense wave at 1.5 mA yields the optimal anxiolytic effects. Additionally, Baihui (GV20) has neural connections with the PVN. EA at Baihui (GV20) exerts its beneficial effects through a PVN-derived OXT-dependent pathway (Fig. 5). The integration of the PVN-OXT pathway, core concepts of TCM, and EA at Baihui (GV20) provides a paradigmatic example of bridging TCM with modern biomedical science. Collectively, these findings may facilitate the elucidation of anatomical circuits linking specific acupoints to distinct brain regions, laying a foundation for the evidence-based optimization of acupuncture therapies for neuropsychiatric disorders.

## Conclusions

Our study reveals that EA applied to GV20 (Baihui) with intermittent 1.5 mA stimulation attenuates anxiety-like behaviour in mice via PVN-OXT dependent circuit. These data establish an anatomically and molecularly defined mechanism for acupoint-specific EA anxiolysis and provide a mechanistic framework for optimising acupuncture-based interventions in anxiety disorders.

## Data Availability

The datasets used and/or analysed during the current study are available from the corresponding author upon reasonable request.

## Abbreviations

AAV: Adeno-associated viral vector
CeA: Central amygdala
DAPI: 2-(4-Amidinophenyl)-6-indolecarbamidine dihydrochloride
dmPAG: Dorsomedial periaqueductal gray
EA: Electroacupuncture
EGFP: Enhanced green fluorescent protein
EPM: Elevated plus maze
HIP: Hippocampus
HPA: Hypothalamic–pituitary–adrenal
LGN: Lateral geniculate nucleus
LH: Lateral hypothalamus
LPB: Lateral parabrachial nucleus
OFT: Open field test
OXT: Oxytocin
OXTR: Oxytocin receptor
PVN: Hypothalamic paraventricular nucleus
PVT: Paraventricular thalamus
